# Aberrant DNA methylation reprogramming during induced pluripotent stem cell generation is dependent on the choice of reprogramming factors

**DOI:** 10.1186/2045-9769-3-4

**Published:** 2014-02-07

**Authors:** Aline C Planello, Junfeng Ji, Vivek Sharma, Rajat Singhania, Faridah Mbabaali, Fabian Müller, Javier A Alfaro, Christoph Bock, Daniel D De Carvalho, Nizar N Batada

**Affiliations:** 1 Campbell Family Cancer Research Institute, Ontario Cancer Institute, Princess Margaret Cancer Centre, University Health Network, Toronto, ON M5G 2 M9 Canada; 2 Department of Morphology, Piracicaba Dental School, University of Campinas, Piracicaba, SP Brazil; 3 Ontario Institute for Cancer Research, Toronto, Ontario M4G 0A3 Canada; 4 Center of Stem Cell and Developmental Biology, Zhejiang University, Hangzhou, Zhejiang Province 310058 China; 5 National Cancer Institute, NIH, Bethesda, MD 20892 USA; 6 Department of Medical Biophysics, University of Toronto, Toronto, ON M5G 2 M9 Canada; 7 Max Planck Institute for Informatics, Campus E1.4, 66123 Saarbrücken, Germany; 8 CeMM Research Center for Molecular Medicine of the Austrian Academy of Sciences, Lazarettgasse 14, 1090 Vienna, Austria; 9 Department of Laboratory Medicine, Medical University of Vienna, Währinger Gürtel 18-20, 1090 Vienna, Austria

## Abstract

The conversion of somatic cells into pluripotent stem cells via overexpression of reprogramming factors involves epigenetic remodeling. DNA methylation at a significant proportion of CpG sites in induced pluripotent stem cells (iPSCs) differs from that of embryonic stem cells (ESCs). Whether different sets of reprogramming factors influence the type and extent of aberrant DNA methylation in iPSCs differently remains unknown. In order to help resolve this critical question, we generated human iPSCs from a common fibroblast cell source using either the Yamanaka factors (OCT4, SOX2, KLF4 and cMYC) or the Thomson factors (OCT4, SOX2, NANOG and LIN28), and determined their genome-wide DNA methylation profiles. In addition to shared DNA methylation aberrations present in all our iPSCs, we identified Yamanaka-iPSC (Y-iPSC)-specific and Thomson-iPSC (T-iPSC)-specific recurrent aberrations. Strikingly, not only were the genomic locations of the aberrations different but also their types: reprogramming with Yamanaka factors mainly resulted in failure to demethylate CpGs, whereas reprogramming with Thomson factors mainly resulted in failure to methylate CpGs. Differences in the level of transcripts encoding DNMT3b and TET3 between Y-iPSCs and T-iPSCs may contribute partially to the distinct types of aberrations. Finally*, de novo* aberrantly methylated genes in Y-iPSCs were enriched for NANOG targets that are also aberrantly methylated in some cancers. Our study thus reveals that the choice of reprogramming factors influences the amount, location, and class of DNA methylation aberrations in iPSCs. These findings may provide clues into how to produce human iPSCs with fewer DNA methylation abnormalities.

**Electronic supplementary material:**

The online version of this article (doi:10.1186/2045-9769-3-4) contains supplementary material, which is available to authorized users.

## Introduction

Ectopic expression of transcription factors highly expressed in ESCs can reprogram somatic cells into pluripotent stem cells [1]. The discovery of this method has generated great excitement because it can potentially provide a patient-specific cell source for cell replacement therapy and can be used to model genetic diseases for drug discovery [2]. iPSCs have now been generated from many different human cell types and various conditions have been identified that increase reprogramming efficiency [3].

Induction of pluripotency by reprogramming factors is accompanied by extensive epigenetic changes in the reprogramming factor recipient cells [4–7]. Methylation of DNA at CpG dinucleotide plays an important role in regulating gene transcription and silencing transposons. Trichostatin A (a histone deacetylase inhibitor) and 5-Azacytidine (a DNA methylation inhibitor) increase reprogramming efficiency suggesting that the resistance of somatic cell chromatin to remodeling is a barrier to reprogramming [4, 5, 8]. Many regions in the genome might have a chromatin state such as heterochromatin that is recalcitrant to remodeling by reprogramming factors. Indeed, a recent study identified hotspots of DNA methylation aberrations in iPSCs; regions flanking centromeres and telomeres were enriched for aberrations [9]. DNA methylation failure at genes specifically expressed in differentiated cells is a well-described type of aberrations present in iPSCs. These aberrations result in expression of differentiated genes in iPSCs that can negatively affect the propensity differentiation into various lineages [10–13]. Furthermore, DNA methylation aberrations have been shown to persist during passaging [11] and differentiation [14, 15]. Consequently, a key concern in the field is that epigenetic aberrations in iPSCs may limit their use in regenerative medicine, disease modeling and drug discovery.

Though new reprogramming factors and combinations have been found, the two most commonly used nowadays are the Yamanaka factors (OCT4, SOX2, KLF4 and cMYC) and the Thomson factors (OCT4, SOX2, NANOG and LIN28). However, the majority of studies have mainly profiled iPSCs derived using a single type of reprogramming factor cocktail (*i.e.* the Yamanaka factors). It is difficult to compare the genome-wide DNA methylation profiles of iPSCs derived using Yamanaka factors against iPSCs (Y-iPSCs) derived using Thomson factors (T-iPSCs) from different studies, since the tissue of origin are different or come from different individuals in each study. Thus it is unknown whether the choice of reprogramming factors influences the extent and the type of DNA methylation aberrations in iPSCs.

In this study, we assessed genome-wide DNA methylation profiles of 15 iPSCs lines made with Yamanaka [16, 17] and Thomson [18] reprogramming factors from a common batch of fibroblasts using the Illumina HumanMethylation450 platform. In agreement with previous studies [10–13, 15] we have found significant levels of recurrent aberrations present in all iPSCs. In addition, we observed DNA methylation aberrations specific for Y-iPSCs and T-iPSCs despite being derived from the same parental cells and undergoing the same conditions and duration in culture. The key finding of our paper is that DNA methylation aberrations in Y-iPSCs are largely due to failure to erase parental methylation while the aberrations in T-iPSCs are largely due to the failure to place ESC specific methylation marks.

## Results

### Genome wide DNA methylation profiles reveal aberrations in iPSCs

**Figure 1:**
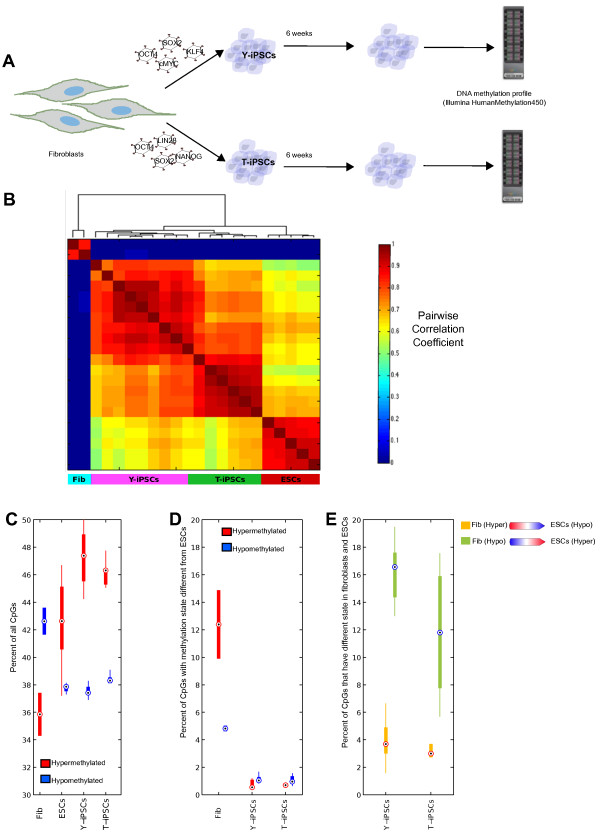
**Experimental design and comparison of DNA methylation profiles of iPSCs and ESCs. A)** Scheme showing the experimental design. Cells were treated identically and 25 days post-infection, colonies were picked randomly and maintained in culture for 6 passages. We profiled DNA methylation during early passage iPSCs to reduce the confounding contribution of negative selection during culture conditions [32], which may mask the differences between these 2 reprogramming factor combinations. **B)** Pair-wise plot shows that all iPSC methylomes are similar to ESC methylomes and significantly different from fibroblast methylomes. The raw methylation data was normalized and background-subtracted using the Illumina Genome Studio software. Intensity values were converted to beta-values where the value of 0 represents unmethylated and the value of 1 represents fully methylated. Fib stands for fibroblasts. **C)** Boxplot of the percentages of highly methylated (red) and lowly methylated (blue) CpGs in fibroblasts, ESCs, Y-iPSCs and T-iPSCs show that their overall levels in iPSCs are largely similar to those in ESCs. Hypermethylated CpGs are defined as those with values > 0.7 and hypomethylated CpGs are defined as those with values < 0.3. Hyper and Hypo stand for hypermethylated and hypomethylated, respectively. **D)** Boxplot of the percentages of highly methylated (red) and lowly methylated (blue) CpGs in fibroblasts, Y-iPSCs and T-iPSCs that are significantly different from ESCs (defined as those with difference in methylation > =0.2) show that only a small proportion of the CpGs in iPSCs are different from those in ESCs. **E)** Boxplot made as in Figure 1D but restricted to only those CpGs that change their methylation state from fibroblasts to ESCs. CpGs hypomethylated in fibroblasts but hypermethylated in ESCs (green) are more likely to be significantly different in iPSCs than CpGs that are hypermethylated in fibroblasts and hypomethylated in ESCs (yellow).

iPSCs were derived from human neonatal foreskin fibroblasts via retroviral infection of reprogramming factors [16, 17]. A common batch of human neonatal foreskin fibroblasts was split into 3 groups (Figure 1A). The first set was directly processed for DNA methylation analysis. The second set was infected with retroviruses encoding the Yamanaka factors, *i.e.* OCT4, SOX2, KLF4 and cMYC reprogramming factors. The third set was infected with retroviruses encoding the Thomson factors, *i.e.* OCT4, SOX2, NANOG and LIN28 reprogramming factors. We selected 9 Y-iPSCs and 6 T-iPSCs that had characteristic ESC-like morphology, express several pluripotency genes and can be stably maintained in culture (Figure 1A; Additional file 1: Figure S1). Genomic DNA from each of the iPSCs was subjected to DNA methylation profiling using the Illumina HumanMethylation450 platform. We augmented our data set with published Illumina HumanMethylation450 methylation data for several male ESCs (SIVF002, SIVF025, SIVF043, SIVF044, SIVF050) [14]. To validate the DNA methylation data obtained by the Illumina HumanMethylation450 platform we performed high throughput sequencing based DNA methylation assessment called reduced representation bisulfite sequencing (RRBS) in one of the Y-iPSCs and one of the T-iPSCs. The overall methylation data from Illumina HumanMethylation450 and RRBS data were concordant (with an average correlation coefficient of 0.8) (Additional file 1: Figure S2). These results are very similar to the Pearson correlation between RRBS and Illumina’s DNA methylation arrays obtained in a different study using the same cutoffs [19] and argue in favor of the reliability of the data obtained in our study.

To determine the concordance of methylation values between parental fibroblast, iPSC and ESC samples, we performed unsupervised consensus clustering [20] with the computed pairwise correlation coefficient of all DNA methylation values and identified 3 DNA methylation clusters (Figure 1B). Cluster 1 formed a particularly tight cluster well separated from the other 2 clusters and was composed by the parental fibroblasts. iPSCs and ESCs comprised clusters 2 and 3, respectively (Figure 1B). This shows that, as expected, the DNA methylomes of iPSCs were distinct from the parental fibroblasts and closer to, but segregated from, the methylomes of ESCs. Fibroblasts had much higher proportion of lowly methylated CpGs than highly methylated CpGs; this trend was opposite in ESCs and iPSCs (Figure 1C). We computed the percentage of CpGs in fibroblasts and iPSCs that are significantly different in methylation state from those in ESCs. Fibroblasts had about 17% of the CpGs that have significantly different levels of methylation than in ESCs, while iPSCs had less than 2% of the CpGs that are different from ESCs (Figure 1D). Interestingly, in fibroblasts, hypermethylated CpGs were more likely to have different methylation state from ESCs than hypomethylated states, while CpGs in iPSCs with methylation values different from ESCs are equally likely to be hypermethylated or hypomethylated (Figure 1D). Finally, CpGs with methylation values significantly different between iPSCs and ESCs are enriched for those that are hypomethylated in fibroblasts compared with ESCs, which means that they fail to get properly methylated in iPSCs (Figure 1E). This result is consistent with previous findings [3].

**Figure 2:**
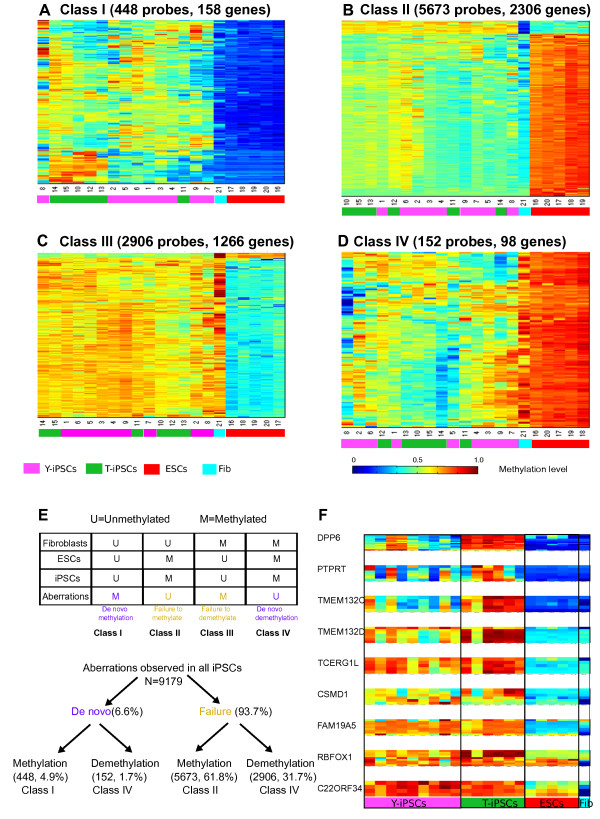
**DNA methylation aberrations that are common in Y- and T-iPSCs. A**. Hypomethylated CpGs in fibroblasts that undergo *de novo* methylation. **B**. Hypomethylated CpGs that fail to be methylated. **C**. Hypermethylated CpGs in fibroblasts that fail to be demethylated. **D**. Hypermethylated CpGs in fibroblasts that get aberrantly methylated. **E**. Summary of the classes of DNA methylation aberrations found in all the iPSCs. **F**. DNA methylation of CpGs at the transcription start site of 9 genes reported to be aberrantly methylated in all iPSCs [15].

In order to identify the CpGs that are differentially methylated between iPSCs and ESCs, we used a DNA methylation difference of more than 0.2 or less than −0.2 and a False Discovery Rate (FDR)-corrected p-value lower than 0.05 as a threshold (Additional file 1: Figure S3). DNA methylation aberrations shared by iPSCs were partitioned into 4 classes: Class I represented *de novo* methylation (defined as methylated regions in iPSCs which are unmethylated in ESCs and in parental cells), Class II represented failed methylation (defined as unmethylated regions in iPSCs which are unmethylated in parental cells but methylated in ESCs), Class III represented failed demethylation (defined as methylated regions in iPSCs which are methylated in parental cells but unmethylated in ESCs); and Class IV represented *de novo* demethylation (defined as unmethylated sites in iPSCs that are methylated in both parental cells and ESCs). Consistent with previous findings, all the iPSCs had DNA methylation aberrations (Figure 2A-E). About 62% of the aberrations were Class II (failed methylation) (Figure 2B) and about 32% of the aberrations were Class III (failed demethylation) (Figure 2D). These results suggest that inadequate change of parental DNA methylation state (also referred to as tissue of origin memory), rather than *de novo* changes in DNA methylation, is the type of aberration shared by all iPSCs regardless of whether the Yamanaka or the Thomson reprogramming factors were used.

A recent study [15], which used a capture-based approach to characterize DNA methylation levels in 17 iPSCs generated derived from several cell types using the Yamanaka factors, identified 9 genes that they found to harbor aberrant DNA methylation regardless of the parental source used. We scrutinized the methylation values of these 9 genes in our dataset and found that only 4 genes (FAM19A5, CSMD1, TCERG1L and TMEM132D) were aberrantly methylated in all the iPSC, while 2 others (DPP6 and TMEM132C) were aberrantly methylated in T-iPSCs only. The DNA methylation levels of the remaining 3 genes (RBFOX1, C22ORF34 and PTPRT) did not show obvious recurrent aberrant methylation (Figure 2F). The discrepancy between their data and our results might be due to differences in the reprogramming protocol, cell culture or the donor cells.

### Y-iPSC and T-iPSC-specific DNA methylation aberrations are of different classes

**Figure 3:**
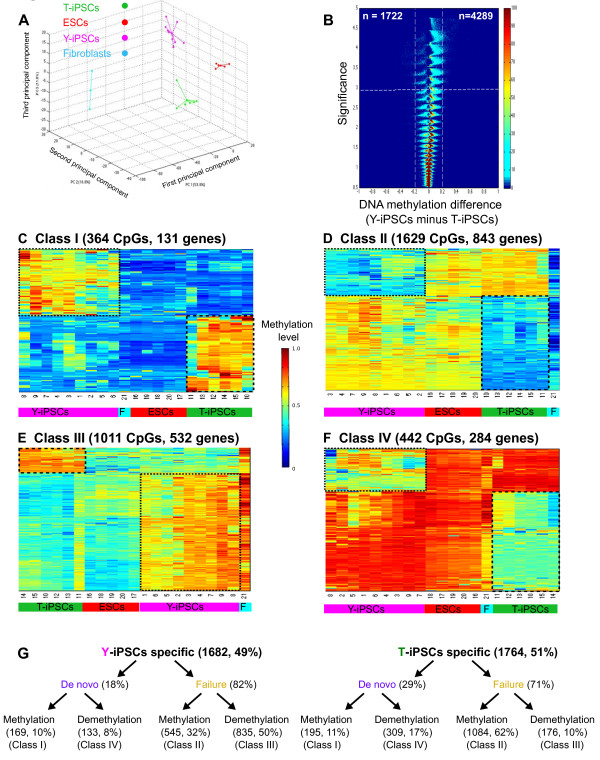
**DNA methylation aberrations that are found only in Y- or T-iPSCs. A**. Principal Component Analysis showing that methylomes of Y-iPSCs, T-iPSCs and ESCs segregate into separate groups. **B**. Volcano plots of all CpG sites analyzed. The beta value difference in DNA methylation between Y-iPSCs and T-iPSCs is plotted on the x-axis, and the *p*-value for a FDR-corrected Wilcoxon signed-rank test of differences between Y-iPSCs and T-iPSCs (shown on − log10 scale) is plotted on the y-axis. CpGs that are significantly different between the 2 subtypes are shown on the upper left corner (significantly hypermethylated in T-iPSCs) and upper right corner (significantly hypermethylated in Y-iPSCs). **C**. CpGs that are hypomethylated in fibroblasts but are aberrantly methylated in iPSCs. **D**. CpGs that are hypomethylated in fibroblasts but fail to acquire methylation in iPSCs. **E**. CpGs that are hypermethylated in fibroblasts but aberrantly demethylated. **F**. CpGs that hypermethylated in fibroblasts but aberrantly gets demethylated in iPSCs. **G**. Summary of the classes of DNA methylation aberrations found only in Y-iPSCs (left) or T-iPSCs (right) but not both.

Though previous studies have identified recurrent DNA methylation alterations in iPSCs when compared to ESCs, no systematic study has been done to determine how the extent and type of DNA methylation aberrations depend on the factors used for reprogramming. Our study was specifically designed to identify these differences. We performed a Principal Component Analysis of global DNA methylation values, and observed that though all the iPSCs are closer to ESCs than fibroblasts, they separated in two distinct groups according to the reprogramming factors used to generate them (Figure 3A). We identified 6,011 CpGs that were differentially methylated between T-iPSCs and Y-iPSCs (1,722 CpGs were hypermethylated in T-iPSCs and 4,289 CpGs were hypermethylated in Y-iPSCs) (Figure 3B; Additional file 2: Table S1). Note that these are not a subset of aberrantly methylated CpGs found in all iPSCs (Figure 2). Among these 6,011 CpGs we observed that in both T-iPSCs and Y-iPSCs they corresponded to all 4 classes of DNA methylation aberrations described above (Figure 3C to G). Interestingly, both T-iPSCs and Y-iPSCs had fewer *de novo* aberrations (Class I and IV) than expected by chance (*p <* 0.001) and more failure aberrations (Class II and III) than expected by chance (*p <* 0.001) (Additional file 1: Figure S4). Moreover, T-iPSCs had substantially more Class II and fewer Class III aberrations than Y-iPSCs (Figure 3G and Additional file 1: Figure S4). Thus, Y-iPSCs mainly suffer from demethylation failure while T-iPSCs mainly suffer from methylation failure. We also observed that *de novo* aberrations (both methylation and demethylation) preferentially present at non-CpG island regions in both Y-iPSCs and T-iPSCs, while failure aberrations (Class II and Class III) are enriched at CpG islands or shores (Additional file 1: Figure S5). Note that approximately 57% (3446 out of 6011 CpGs identified in Figure 3B) of the CpGs that showed recurrent differences between Y-iPSCs and T-iPSCs did not fall into Class I or IV. Interestingly, aberrantly methylated CpGs (Class I) in Y-iPSCs and T-iPSCs were enriched at transcription start sites (TSS) (Additional file 1: Figure S6).

### Enrichment of aberrations at reprogramming factor-binding regions suggests targeting as a potential cause of DNA methylation aberrations in Y-iPSCs and T-iPSCs

**Figure 4:**
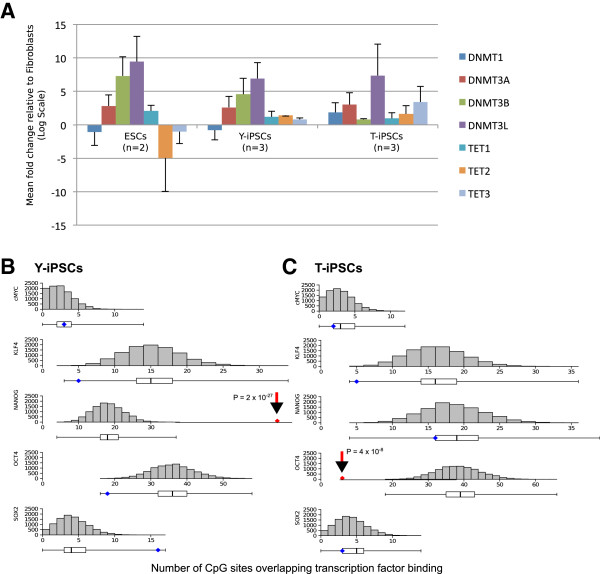
**Levels of DNA methylation and demethylation enzymes and overlap of aberrantly methylated regions with genomic binding sites of reprogramming factors. A**. Expression levels of enzymes involved in methylation and demethylation in pluripotent cells measured using qPCR. Data is log2 transformed and normalized to levels of these enzymes in human fibroblasts. Values shown are the mean of the following number of samples: n = 2 (ESCs), n = 3 (Y-iPSCs) and n = 3 (T-iPSCs). **B** and **C**. Random sampling simulation to determine the distribution of the expected number of genes that are targets of the indicated reprogramming factor and have aberrant DNA methylation CpGs overlapping the binding site. The histograms and box plots represent the null distribution for the overlap between random sampling DNA methylation aberrations and each transcription factor binding sites. The diamonds represent the experimentally observed number of overlap between Y-iPSCs **(B)** or T-iPSCs **(C)** DNA methylation aberrations and each transcription factor binding sites. Red diamonds highlight a number of overlaps that fall completely outside of the null distribution.

Among other possibilities, differences in the classes of aberrations in Y-iPSCs and T-iPSCs might be due to different levels of DNA methylation and demethylation enzymes during reprogramming or it might be due to different targeting of these enzymes to the genomic regions containing the aberrations. To determine whether the levels and the targeting of DNA methylation machinery may contribute to the observed aberrations, we first tested whether the levels of DNA methylation and demethylation enzymes are aberrant. We found that the level of DNMT3b was lower in T-iPSCs than in ESCs or Y-iPSCs, which may potentially contribute to the *de novo* methylation defects in Y-iPSCs (Figure 4A). Moreover, TET3 was higher in T-iPSCs than ESCs or Y-iPSCs and may potentially contribute to excessive demethylation in Y-iPSCs (Figure 4A).

To determine if the targeting of DNA methylation machinery to specific loci by the reprogramming factors might contribute to the observed aberrations we analyzed the methylation defects at genes targeted by the reprogramming factors. We analyzed published ChIP-seq data and quantified the enrichment of aberrations at regions bound by reprogramming factors in ESCs [21] (Additional file 3: Table S2). We observed that CpGs with an aberrant DNA methylation (Class I and II) in Y-iPSCs are significantly (*P* = 2 x 10^-27^) enriched for the NANOG binding sites in ESCs (Figure 4B). Moreover, CpGs that are aberrantly methylated (Class I and II) in T-iPSCs are significantly (*P* = 4 x 10^-8^) depleted at OCT4 bound regions (Figure 4C). The statistically significant increase in aberrations in Y-iPSCs at CpGs within the genomic regions bound by NANOG suggests that some of the CpG aberrations might be a consequence of inadequate targeting of DNA methyltransferase or the DNA demethylation machinery to CpG sites and may contribute to differences between the methylomes of Y-iPSCs and T-iPSCs (Figures 1B and 3A). The list of all genes whose TSS contain one or more aberrantly methylated CpGs are provided in Table 1.

**Table 1:** **List of Genes with aberrant promoter DNA methylation**

iPSC type	Gene name	Position relative to TSS (base pairs)	Type of DNA methylation aberration	Overlapping TF binding site	Delta Beta (Y-iPSCs – T-iPSCs)
Y-iPS	L1TD1	3887	failure to demethylate	SOX2, NANOG	0.205552761
Y-iPS	ZNF202	−849	failure to demethylate	SOX2, NANOG	0.253028526
Y-iPS	VRTN	−7	failure to demethylate	NANOG	0.204685702
Y-iPS	ZNF281	−893	failure to demethylate	NANOG	0.217824711
Y-iPS	PCBP1-AS1	1655	failure to demethylate	NANOG	0.260023778
Y-iPS	CELF2	−602	failure to demethylate	NANOG	0.201570322
Y-iPS	GCLC	2741	failure to demethylate	NANOG	0.209894956
Y-iPS	CCDC85A	−1483	failure to demethylate	NANOG	0.231654822
Y-iPS	GPC6	−1912	failure to demethylate	NANOG	0.2007506
Y-iPS	RNF175	1925	failure to demethylate	NANOG	0.24800605
Y-iPS	LAPTM4B	2519	failure to demethylate	NANOG	0.231217277
Y-iPS	DUSP5	2071	failure to demethylate	NANOG	0.254193033
Y-iPS	TDGF1	1259	failure to demethylate	NANOG	0.250850333
Y-iPS	ZFP36L1	−544	failure to demethylate	KLF4	0.310706089
Y-iPS	CDC16	−199, −212	spurious methylation	OCT4	0.362236745, 0.417741778
Y-iPS	ADAR	−226	spurious methylation	OCT4	0.201398056
Y-iPS	RPS23	3852	failure to demethylate	OCT4	0.234123761
Y-iPS	ZBTB44	−1063, −1044	failure to demethylate	OCT4	0.234429233, 0.308618094
Y-iPS	KIF3C	−433, −422	failure to demethylate	OCT4	0.348975978, 0.365098844
Y-iPS	HORMAD2	−354, −363	failure to demethylate	OCT4	0.233920207, 0.252708599
Y-iPS	PSMA8	23	failure to demethylate	cMYC	0.238296222
Y-iPS	PLEKHG6	−189	failure to demethylate	cMYC	0.203390178
T-iPS	TNFSF8	−1308	failure to methylate	NANOG	0.387689344
T-iPS	FLJ31485	3444	failure to demethylate	NANOG	−0.340429706
T-iPS	KCNH2	53	failure to methylate	KLF4	0.246395267
T-iPS	TMEM132C	−365, −905	spurious methylation	KLF4, OCT4	−0.327425161,−0.3128155
T-iPS	WDR45L	−469, −500	failure to methylate	OCT4	0.291398672, 0.308650794
T-iPS	PABPC1L2A	1760	failure to methylate	cMYC	0.272031639

Genes within 5 kilobase window around TSS that were bound by cMYC, OCT4, SOX2, NANOG and KLF4 in human ESCs are shown. TF stands for transcription factor.

We used Genomic Regions Enrichment of Annotations Tool [22] to determine enrichment of specific classes of genes. Genomic regions with aberrant DNA methylation patterns in Y-iPSCs are enriched for genes with roles in stem cell maintenance (which are normally hypermethylated in fibroblasts), while genomic regions with aberrant DNA methylation patterns in T-iPSCs are enriched for lineage specific genes (which are normally hypomethylated in fibroblasts) (Table 2).

**Table 2:** **Gene ontology of genes associated with differentially methylated regions**

iPS type	GO Biological process	P-value	FDR Q-value	Fold enrichment
Y-iPSCs	Regulation of glial cell proliferation	5.72 × 10^-13^	5.01 × 10^-9^	8.2103
Y-iPSCs	Intestinal epithelial cell differentiation	2.98 × 10^-11^	4.35 × 10^-8^	10.7333
Y-iPSCs	Stem cell development	6.19 × 10^-6^	9.04 × 10^-4^	2.247
Y-iPSCs	Stem cell maintenance	6.81 × 10^-6^	9.63 × 10^-4^	2.2958
T-iPSCs	Eye development	3.81 × 10^-11^	6.68 × 10^-8^	6.5284
T-iPSCs	Spinal cord development	3.68 × 10^-9^	3.58 × 10^-6^	2.4715
T-iPSCs	Limb bud formation	6.50 × 10^-6^	1.09 × 10^-3^	3.1573

GREAT tool [22] was used annotate genes with aberrant methylation in their transcription start site.

### Relationship between DNA methylation aberrations in iPSCs and DNA methylation aberration in cancer

**Figure 5:**
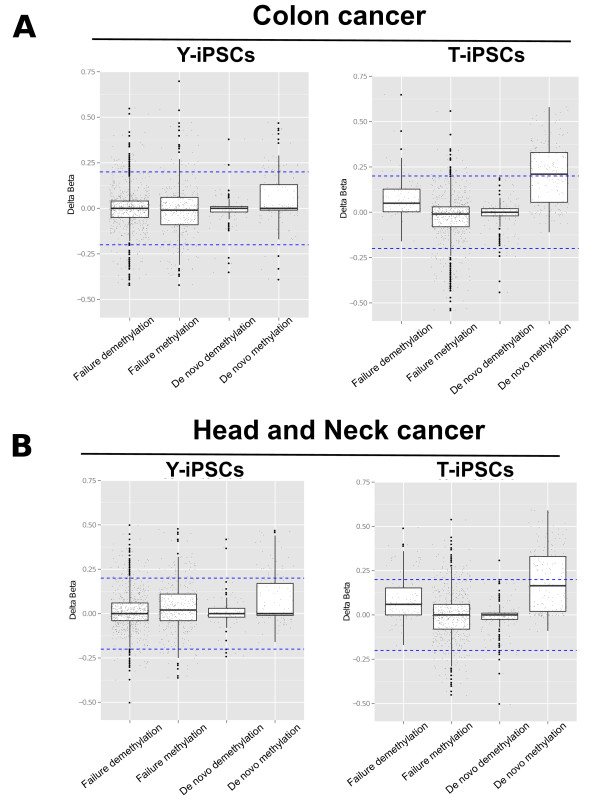
**Cancer versus Normal profile of Y-iPSCs and T-iPSCs aberrantly methylated regions.** DNA methylation difference (delta beta value) between cancer patients and matched normal tissue of each CpG site identified as aberrantly methylated in Y-iPSCs (left panels) or T-iPSCs (right panels). The DNA methylation data was obtained from the TCGA depository (http://tcga-data.nci.nih.gov) for **(A)** Colon adenocarcinoma – COAD (258 tumor samples and 38 normal samples), **(B)** Head and neck squamous cell carcinoma – HNSC (310 tumor samples and 50 normal samples).

One major concern related to use iPSCs for regenerative medicine is the increased tumorigenicity of iPSCs. To gain further insight into the differences in the type of DNA methylation aberrations found in Y-iPSCs or T-iPSCs, we tested whether the differentially methylated CpGs are also aberrantly methylated in cancers. We obtained publicly available DNA methylation data generated by The Cancer Genome Atlas consortium [23]. We evaluated the DNA methylation difference (delta beta value) between cancer patients and matched normal tissue of each CpG site identified as aberrantly methylated in Y-iPSCs (Figure 5, left panels) or T-iPSCs (Figure 5, right panels). We analyzed 6 cancer types: bladder urothelial carcinoma (Additional file 1: Figure S7), breast invasive carcinoma (Additional file 1: Figure S7), colon adenocarcinoma (Figure 5A), head and neck squamous cell carcinoma (Figure 5B), lung adenocarcinoma (Additional file 1: figure S7), lung squamous cell carcinoma (Additional file 1: Figure S7). We observed that CpG sites that exhibited Class I aberrations in T-iPSCs were enriched (*P* < 0.05) among Class I aberrations in colon adenocarcinoma and head and neck squamous cell carcinoma (Figure 5). It is not obvious if genes present within the regions proximal to Class I aberrations shared between T-iPSCs and the 2 cancers play a functional role or have features that would make them susceptible to spurious methylation.

## Discussion

The determinants and the temporal order of epigenetic changes during reprogramming to pluripotency are poorly understood. It is clear that to acquire pluripotency, reprogramming cells must erase differentiation-specific epigenetic marks to return to the state that defines the uncommitted pluripotent embryonic stem cell state [24].

Several studies have revealed that many iPSCs that pass stringent criteria for pluripotency still have non-random DNA methylation differences as compared to ESCs which persist during passaging [11, 14] and differentiation [14, 15]. Incomplete erasure of DNA methylation marks during the reprogramming of somatic cells into pluripotent stem cells is thought to underlie the phenotypic differences such as the higher propensity of iPSCs to differentiate into the lineage from which they were derived, a phenomenon referred to as epigenetic memory [9–15].

In this study, we observed that despite being generated from a common parental cell type, the epigenome of iPSCs harbor distinct DNA methylation aberrations depending on the sets of reprogramming factors used. In agreement with previous reports, our results also show that DNA methylation aberrations are dominated by hypomethylation (62%); however, we have found that a significant proportion of them are also hypermethylated as compared to ESCs (32%). Overall, however, we have found very few instances of aberrant *de novo* methylation or demethylation in iPSCs; rather, the vast majority of the aberrantly methylated sites are result of methylation and demethylation failure at various sites within the parental epigenome during reprogramming.

A key finding of our study is that the class of DNA methylation aberrations in Y-iPSCs and T-iPSCs differ significantly. Y-iPSCs specific DNA methylation aberrations are mainly characterized by demethylation failure, suggesting that Yamanaka factor-driven cellular changes during reprogramming result in deficiency in demethylation. In contrast, T-iPSCs specific DNA methylation aberrations are mainly characterized by DNA methylation failure, suggesting that Thomson factor-driven cellular changes during reprogramming result in deficient DNA methylation. As the silencing of differentiation genes in somatic cells is essential for achieving the pluripotent state [24], the insufficient methylation activity in driven by the Thomson factors compared to Yamanaka factors might explain why efficiency of reprogramming with the former is routinely lower than when using Yamanaka factors. Given that T-iPSCs are hypomethylated relative to ESCs and Y-iPSCs are hypermethylated relative to ESCs, it might be the case that the observed epigenetic memory or bias of iPSCs to differentiate into parental lineage [10, 11], which is thought to arise due to incomplete silencing of somatic genes, may be higher in T-iPSCs than in Y-iPSCs.

The differences in methylation and demethylation activity during reprogramming (Figure 4A) with Yamanaka and Thomson reprogramming factors can provide a rationale for selecting reprogramming factors and reprogramming efficiency-enhancing epigenetic drugs. In particular, Thomson factors might be a better at reprogramming cell types that have highly methylated genomes, while the Yamanaka factors might be better suited for reprogramming of somatic cells that have large proportion of open chromatin that needs to be silenced during reprogramming. We speculate that reagents that enhance DNA demethylation may prove more helpful in overcoming the failed-type DNA demethylation aberrations in Y-iPSCs reprogramming than in T-iPSCs reprogramming, since the latter is more efficient in inducing DNA demethylation.

We note that the approach used for DNA methylation profiling is based on bisulfite conversion and therefore cannot distinguish between 5-methylcytosine (5-mC) and 5-hydroxymethylcytosine (5-hmC). This means that the CpGs identified as methylated can either be 5-mC or 5-hmC. This technical limitation does not invalidate our finding that Y-iPSCs aberrations are mainly DNA demethylation failure. Even if some of the sites classified as demethylation failure are actually 5hmC (instead of 5mC), the same sites are completely unmethylated in T-iPSCs. Therefore, Y-iPSCs is indeed failing to demethylate these sites, either by maintaining 5mC or by converting to 5hmC but not been able to further convert to unmethylated cytosine.

**Figure 6:**
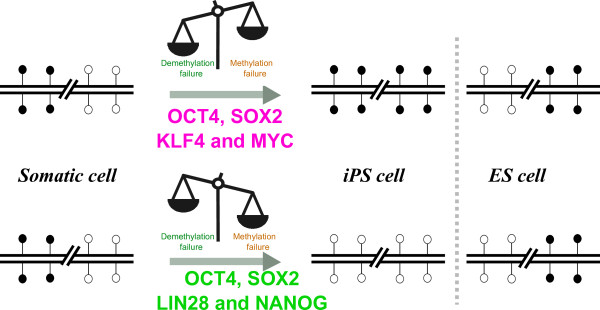
**Model relating reprogramming factors and types of DNA methylation aberrations.** A hypothetical genomic region showing four CpGs. White disk represent unmethylated CpG and black disk represent methylated CpG. Reprogramming with the Yamanaka factors leads to proper methylation but failure to demethylate, leading to enrichment of DNA demethylation aberrations in these iPSCs relative to ESCs. Similarly, reprogramming with the Thomson factors leads to proper demethylation but failure to methylate, leading to enrichment of DNA methylation aberrations in these iPSCs relative to ESCs.

In conclusion, our study has revealed that different reprogramming factor combinations lead to differences in the type and extent of DNA methylation aberrations observed in iPSCs (Figure 6). It is possible that the differences in the DNA methylation landscape between Y-iPSCs and T-iPSCs may lead to subtle phenotypic consequences even when starting with the exact same donor cell type. We suggest that the differences between T-iPSCs and Y-iPSCs highlighted above might be advantageously used for achieving optimal reprogramming of various donor cell types depending on their overall levels of DNA methylation of their genome.

## Methods and materials

### Cell culture

Human neonatal foreskin fibroblasts (ATCC, Manassa, VA) were cultured in growth medium consisting of DMEM (Invitrogen, Carlsbad, CA) supplemented with 10% fetal calf serum (Hyclone Laboratories, Mississauga, ON) and 1 mM L-glutamine (Invitrogen). iPSCs were maintained on a Matrigel (BD Biosciences, Mississauga, ON) -coated plate in complete mTeSR1 medium (Stemcell Technologies, Vancouver, BC) as previously described [25].

### Virus production

Four moloney-based retroviral vectors (pMXs) containing the human complementary DNAs (cDNAs) of OCT4, SOX2, KLF4, cMYC, NANOG and LIN28 were obtained from Addgene (Cambridge, MA). These plasmids were transfected into the 293GPG packaging cell line that incorporated pMD.gagpol and tetracycline-inducible VSV-G plasmids to generate high titer retroviruses as previously described [26]. Viral supernatant was collected 48, 72 and 96 hours post-transfection and filtered by 0.45 μm syringe filters.

### Generation of iPSCs

iPSCs were generated as previously described [16]. Briefly, 4 × 10^5^ fibroblasts were seeded in gelatin-coated 100 mm dishes in fibroblast medium and were infected twice by OCT4, SOX2, KLF4 and cMYC or OCT4, SOX2, NANOG and LIN28 transgene containing retroviruses during a 48 hours period after seeding fibroblasts. Approximately 24 hours after second viral infection, cells were switched to ESC media consisting of knockout DMEM supplemented with 20% knockout serum replacement, 1 mM L-glutamine, 1% non-essential amino acid, 0.1 mM ß-mercaptoethanol and 10 ng/ml human basic fibroblast growth factor (bFGF; Invitrogen). Approximately 3 to 4 weeks post-infection, newly formed colonies with ESC-like morphology were picked.

### DNA methylation profiling

Genomic DNA samples (1 ug each) were bisulfite converted using the Zymo EZ DNA methylation kit (Zymo Research, Orange, CA, USA; catalog #D5002) according to the manufacturer’s instructions. All cell lines passed bisulfite conversion quality control and were subsequently processed for the Illumina Infinium DNA methylation platform (HumanMethylation450 BeadChip). A beta value (β) of 0–1.0 was reported for each CpG site (methylation ranging from 0% to 100%, respectively). β values were calculated by subtracting background and taking the ratio of the methylated signal intensity to the sum of both unmethylated (U) and methylated (M) signals: M/(M + U).

The McGill University Genome Quebec Innovation Centre in Montreal performed the Infinium methylation assays in accordance with the manufacturer’s instructions. The assay information is available at http://www.illumina.com. The Infinium array cover 482,421 CpG sites of approximately 28 million sites in the human genome and covers 99% of RefSeq genes, averaging 17 CpG sites per gene. It covers 96% of human CpG islands, with additional coverage in island shores and the regions flanking them.

### Preprocessing of Illumina HumanMethylation450 data

Data were normalized and background corrected using GenomeStudio. Briefly, the red and green signals were normalized by multiplying the green signal by the product of the red channel control value divided by the green channel control value. Control values were obtained from the control profile file. Background subtraction was performed from both channels using the negative probe control values.

### Identification of differentially methylated CpGs

To identify differentially methylated CpGs in iPSCs, we used a well-accepted method and threshold [27, 28]. We calculated the DNA methylation difference and FDR-corrected Wilcoxon signed-rank test of differences between ESCs and iPSCs. We established a DNA methylation difference of more than 0.2 or less than −0.2 and an FDR-corrected *p*-value lower than 0.05 as a threshold to define a differentially methylated probe (Additional file 1: Figure S3). In addition, to identifying whether each of the differentially methylated CpGs in iPSCs falls under each theoretical class DNA methylation aberration, we divided the CpGs in 4 groups: 1) heavily methylated in ESCs and fibroblasts; 2) heavily unmethylated in ESCs and fibroblasts; 3) significantly hypomethylated in fibroblasts compared to ESCs; and 4) significantly hypermethylated in fibroblasts compared to ESCs. Statistical significance of the CpGs was evaluated using 9 independent Y-iPSCs and 6 independent T-iPSCs, all generated from the same batch of fibroblasts. Significant differences in DNA methylation levels in Y-iPSCs and T-iPSCs were determined for each probe using Wilcoxon rank sum test followed by an FDR correction, using the Benjamini and Hochberg approach, in order to correct for multiple tests. The raw p-value and the FDR-corrected p-value were calculated for all CpGs in the assay and presented as a Density Volcano Plot. The mean beta value across each group was computed and then subtracting from the beta value of each probe to generate the beta value difference between the two groups. All the statistical tests were performed in Matlab using the statistical and bioinformatics toolboxes.

### Computation of enrichment of aberrations at regions bound by reprogramming factors in ESCs

We then used permutation testing (10,000 permutations, without replacement) to generate null distributions from randomized differentially methylated CpGs (aberrant in T-iPSCs or aberrant in Y-iPSCs) across all the CpGs in the array. We then counted how many times each of this random null distribution overlaps with the experimentally ascertained transcription factor-binding peaks and calculated the exact permutation p-values [29].

### Reduced Representation Bisulfite Sequencing (RRBS)

RRBS was performed according to a previously published protocol [30, 31] with the following modifications: 15 ng of genomic DNA from Y-iPSCs and T-iPSCs was the initial input for MSPI digestion. For ligation step the adapters from TruSeq Kit (Illumina) were used diluted 1:4 and 1.5 μl added in the reaction. The incubation time for ligation was 24 hours. For the first amplification step, after bisulfite conversion and before fragment selection, reaction was performed in 25 μl containing 1x Kapa HiFi HotStart Uracil + Ready mix (MA, USA), 3 μl of Cocktail PCR primer (TruSeq kit) and 8 μl of bisulfite converted DNA (Sigma). The amplification program consisted of an initial denaturation step at 98°C during 45 seconds and 6 cycles of: 15 seconds at 98°C, 30 seconds at 60°C, 30 seconds at 72°C, and a final elongation step of 1 minute at 72°C. Fragment size selection was performed with 3% Nusieve 3:1 agarose 0.5x TBE gel and the gel ladder was stained with 1:20,000 dilution of RedSafe (Chembio, UK). The gel was cut between 150 and 400 base pairs. Gel extraction was performed with Qiagen Minelute kit in 2 columns and eluted in 10 μl each. Final library was prepared in 50 μl containing 1 μl of Kapa Hifi HotStart DNA polymerase (1U/μl) (Kapa biosystems, MA, USA), 10 μl of 5× Kapa HiFi fidelity Buffer, 1.5 μl of Kapa dNTP mix (10 nm each), 6 μl of TruSeq Cocktail PCR primer and 20 μl of size selected DNA fragments. The amplification program consisted of an initial denaturation step at 95°C during 2 minutes and 12 cycles of: 20 seconds at 98°C, 30 seconds at 60°C, 30 seconds at 72°C, and a final elongation step of 1 minute at 72°C. The final library was purified using Agencourt AMpure XP magnetic beads (Beckman Coulter) according to the manufacturer’s protocol.

### Comparison of Illumina HumanMethylation450 and sequencing based DNA methylation profiles

In RRBS, DNA is digested with Msp1 restriction enzyme (which is methylation insensitive) and 200 to 400 pair fragments are extracted, bisulfite converted and sequenced. The sequence reads were aligned to the genome using the Novoalign (http://novocraft.com) aligner (onto the *in silico* converted genome reference) and only uniquely aligning reads were retained. Methylation levels at CpG with a minimum of 5-fold coverage were computed. RRBS computed methylation levels and Illumina HumanMethylation450 methylation levels can be compared directly and without normalization because both methods measure absolute DNA methylation levels. For a total of 2,710 single CpGs that were covered by both an Illumina HumanMethylation450 probe and at least five RRBS reads in Y-iPSCs, we observed a Pearson correlation of 0.79 (Additional file 1: Figure S2). For T-iPSCs, we obtained a total of 2,398 single CpGs that were covered by both methods and a Pearson correlation of 0.81 (Additional file 1: Figure S2).

### RNA extraction

RNA was purified from parental fibroblasts, Y-iPSCs, T-iPSCs, and ESCs using Trizol (Invitrogen, CA, USA) protocol. Using SuperScript III (Invitrogen) 2 μg of RNA were taken to RT reaction in a final volume of 20 μl. The cDNA was diluted to a final volume of 50 μl and 1 μl was used for the qPCR.

### qPCR conditions

All qPCR reactions were carried out in an Applied Biosystems One Step thermal cycler (CA, USA). The reactions were performed in 20 μl final volume containing 1 × SYBR Select Mastermix (Applied Biosystems CA, USA), primers at concentration of 250 nM. The reactions were performed in experimental and biological triplicate. The amplification program consisted of an initial denaturation step at 95°C during 5 minutes and 40 cycles of: 20 seconds at 95°C, 30 seconds at 60°C, 45 seconds at 72°C, and a final elongation step of 1 minute at 72°C. After the amplification protocol, melting curves were performed in order to analyze the specificity of the amplified fragments, by measuring fluorescence signal at incrementing temperatures of 0.3°C, from 65°C to 95°C. The internal control was GAPDH gene.

### Data availability

The Illumina HumanMethylation450 is available online at Gene Expression Omnibus (http://www.ncbi.nlm.nih.gov/geo/) accession number GSE54115.

## Electronic supplementary material

Additional file 1: **Characterization of iPS lines used in the paper.** (A) Phase contrast images showing colony morphology for all the Y-iPS (OSKM) and T-iPS (OSLN) lines used in this study. All iPS cells were stably maintainable in culture till passage 12 (after which they were frozen). Scale bar, 350 μm. (B) qPCR analysis of pluripotency markers. **Figure S2.** Validation of Illumina Infinium HumanMethylation450 platform by Reduced Representation Bisulfite Sequencing (RRBS). Correlation between the DNA methylation levels accessed by HumanMethylation450 and RRBS in a Y-iPS (A) and a T-iPS (B) sample. **Figure S3.** The beta value difference in DNA methylation between iPS cells and ES cells is plotted on the x axis, and the p value for a FDR-corrected Wilcoxon signed-rank test of differences between iPS cells and ES cells is plotted on the y axis. **Figure S4.** Observed versus expected ratio for the four classes of aberrations to the DNA methylation pattern. **Figure S5.** Observed versus expected ratio for the position relative to a CpG Island of each class of aberrations to the DNA methylation pattern. (A) Schematic distribution of CpG islands, shores, shelfs and open sea. Ratio between the observed number of CpGs in the spurious demethylation class (B), spurious methylation class (C), failure to methylate class (D), failure to demethylate class (E) versus the randomly expected number of CpGs. **Figure S6.** Region-Gene association graph for the position relative to a TSS of T-iPS and Y-iPS-specific aberrations. (A) Y-iPS specific aberrations. (B) T-iPS specific aberrations. **Figure S7.** Cancer versus Normal profile of Y-iPS and T-iPS aberrantly methylated regions. DNA methylation difference between cancer patients and matched normal tissue of each CpG site identified as aberrantly methylated in Y-iPS cells (left panels) or T-iPS cells (right panels). The DNA methylation data was obtained from the TCGA depository (http://tcgadata.nci.nih.gov) for (A) Bladder Urothelial Carcinoma – BLCA (171 tumor samples and 19 normal samples), (B) Breast invasive carcinoma – BRCA (613 tumor samples and 97 normal samples), (C) Lung adenocarcinoma – LUAD (409 tumor samples and 32 normal samples) and (D) Lung squamous cell carcinoma – LUSC (252 tumor samples and 42 normal samples). (PDF 2 MB)

Additional file 2: **DNA methylation data for CpGs differentially methylated between Y-iPS and T-iPS.** (XLS 5 MB)

Additional file 3: **List of genomic binding sites of reprogramming factors in H1 ES cells that overlap with aberrantly methylated regions.** (XLS 14 KB)
