# Supplementary material for: Aberrant DNA methylation reprogramming during induced pluripotent stem cell generation is dependent on the choice of reprogramming factors
Source: Cell Regen. 2014 Feb 7;3:4. doi: 10.1186/2045-9769-3-4 (PMC4230737; doi:10.1186/2045-9769-3-4)
Supplement: Supplementary file 1 — Additional file 1: Characterization of iPS lines used in the paper. (A) Phase contrast images showing colony morphology for all the Y-iPS (OSKM) and T-iPS (OSLN) lines used in this study. All iPS cells were stably maintainable in culture till passage 12 (after which they were frozen). Scale bar, 350 μm. (B) qPCR analysis of pluripotency markers. Figure S2. Validation of Illumina Infinium HumanMethylation450 platform by Reduced Representation Bisulfite Sequencing (RRBS). Correlation between the DNA methylation levels accessed by HumanMethylation450 and RRBS in a Y-iPS (A) and a T-iPS (B) sample. Figure S3. The beta value difference in DNA methylation between iPS cells and ES cells is plotted on the x axis, and the p value for a FDR-corrected Wilcoxon signed-rank test of differences between iPS cells and ES cells is plotted on the y axis. Figure S4. Observed versus expected ratio for the four classes of aberrations to the DNA methylation pattern. Figure S5. Observed versus expected ratio for the position relative to a CpG Island of each class of aberrations to the DNA methylation pattern. (A) Schematic distribution of CpG islands, shores, shelfs and open sea. Ratio between the observed number of CpGs in the spurious demethylation class (B), spurious methylation class (C), failure to methylate class (D), failure to demethylate class (E) versus the randomly expected number of CpGs. Figure S6. Region-Gene association graph for the position relative to a TSS of T-iPS and Y-iPS-specific aberrations. (A) Y-iPS specific aberrations. (B) T-iPS specific aberrations. Figure S7. Cancer versus Normal profile of Y-iPS and T-iPS aberrantly methylated regions. DNA methylation difference between cancer patients and matched normal tissue of each CpG site identified as aberrantly methylated in Y-iPS cells (left panels) or T-iPS cells (right panels). The DNA methylation data was obtained from the TCGA depository (http://tcgadata.nci.nih.gov) for (A) Bladder Urothelial Carcinoma – BLCA ( [file 13619_2013_19_MOESM1_ESM.pdf]

A

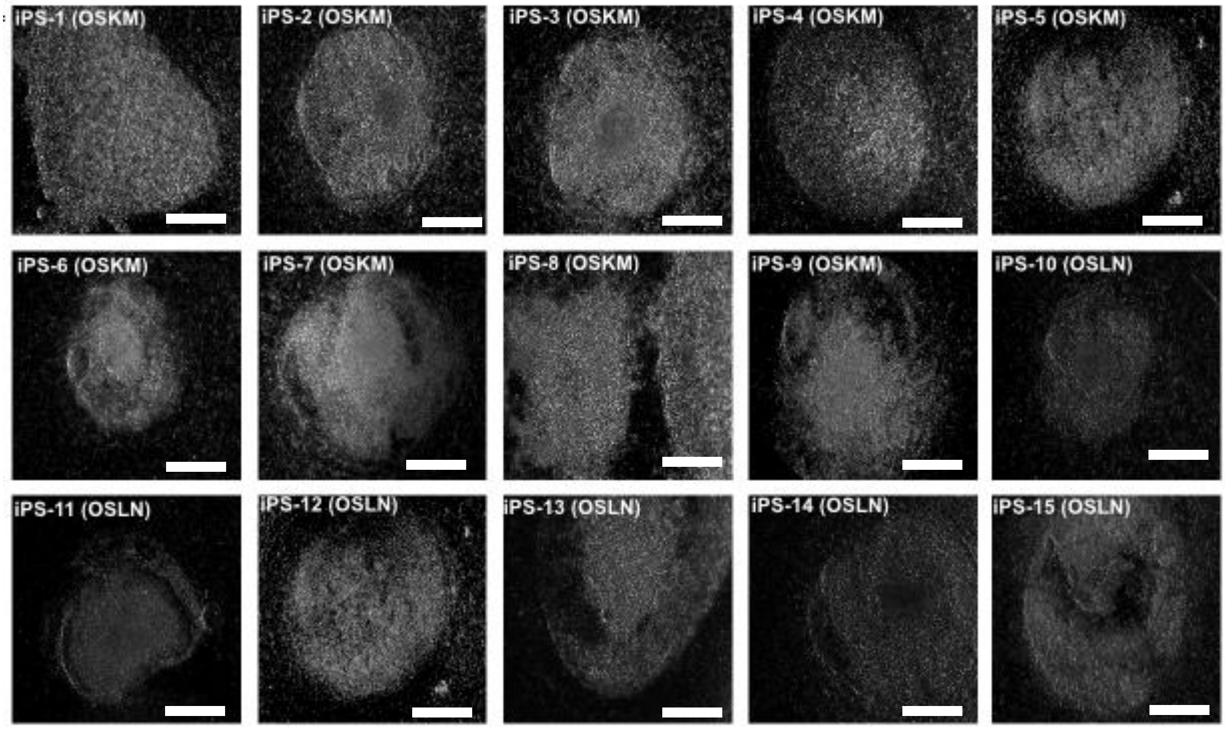

B

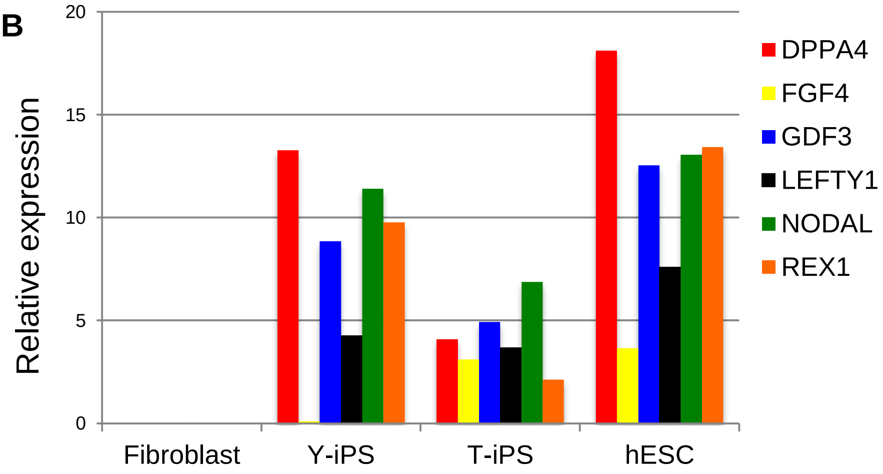

Figure S1. **Characterization of iPS lines used in the paper.**
(A) Phase contrast images showing colony morphology for all the Y-iPS (OSKM) and T-iPS (OSLN) lines used in this study. All iPS cells show characteristic ES-like morphology and at the time of writing of this paper, they were stably maintainable in culture till passage 12 (after which they were frozen). (B) qPCR analysis of pluripotency markers. Relative expression levels were normalized to GAPDH (a house keeping gene). Mean of the normalized expression levels of all the Y-iPS and all the T-iPS is shown. Embryonic Stem Cell line hES-2 was used as a positive control of pluripotent line. Scale bar, 350 μm

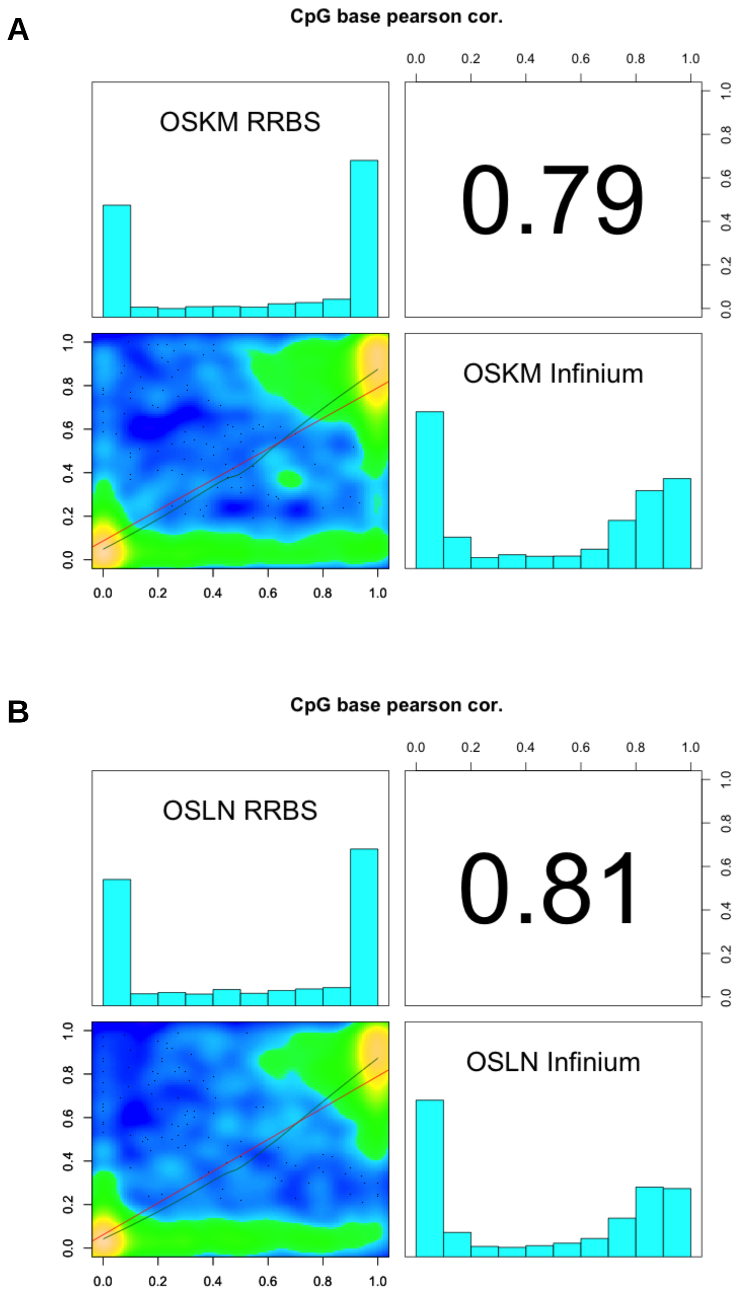

Figure S2. **Validation of Illumina Infinium HumanMethylation450 platform by Reduced Representation Bisulfite Sequencing (RRBS).**

Correlation between the DNA methylation levels accessed by HumanMethylation450 and RRBS in a Y-iPS (A) and a T-iPS (B) sample. The upper-left plot is a histogram for percent methylation distribution by RRBS and the bottom-right plot is a histogram for percent methylation distribution by HumanMethylation450 array. The upper-right plot is the pearson correlation coefficient between RRBS and HumanMethylation450 array. The bottom-left is a cloud-plot of the the DNA methylation levels measured by RRBS (X-axis) and by HumanMethylation450 array (Y-axis).

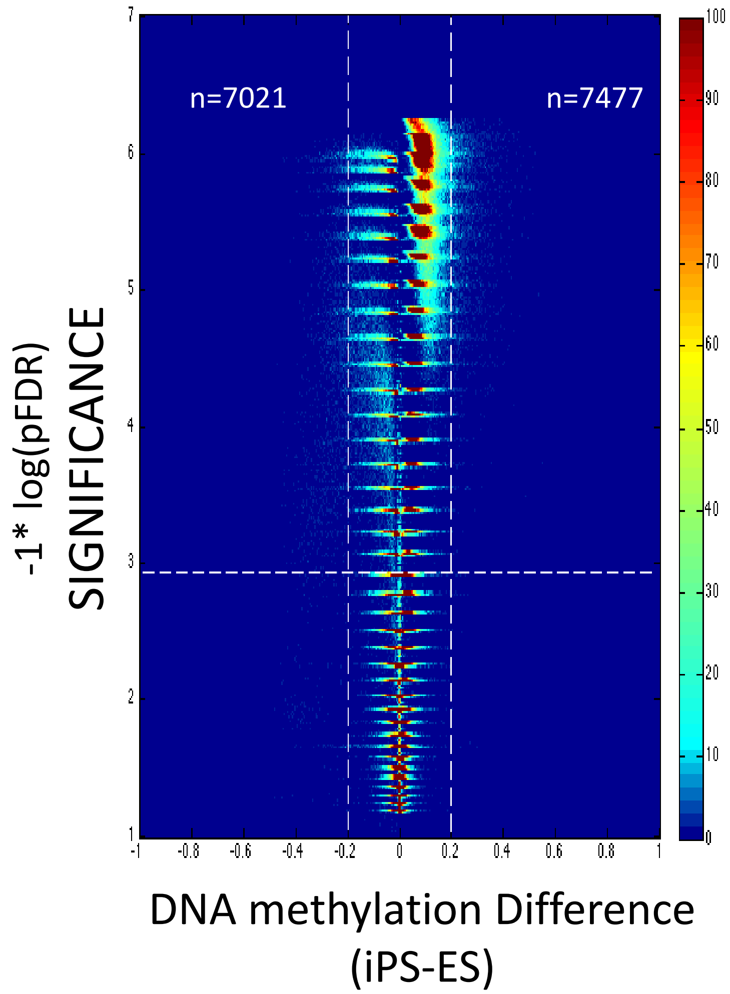

Figure S3 | **DNA Methylation differences between iPS cells (Yamanaka factor derived iPS, Thompson derived iPS) versus ES cells.** Volcano plots of all CpG sites analyzed. The beta value difference in DNA methylation between iPS cells and ES cells is plotted on the x axis, and the p value for a FDR-corrected Wilcoxon signed-rank test of differences between iPS cells and ES cells (−1∗ log10 scale) is plotted on the y axis. Probes that are significantly different between the two subtypes are shown on the upper left corner (significantly hypermethylated in ES cells) and upper right corner (significantly hypermethylated in iPS cells).

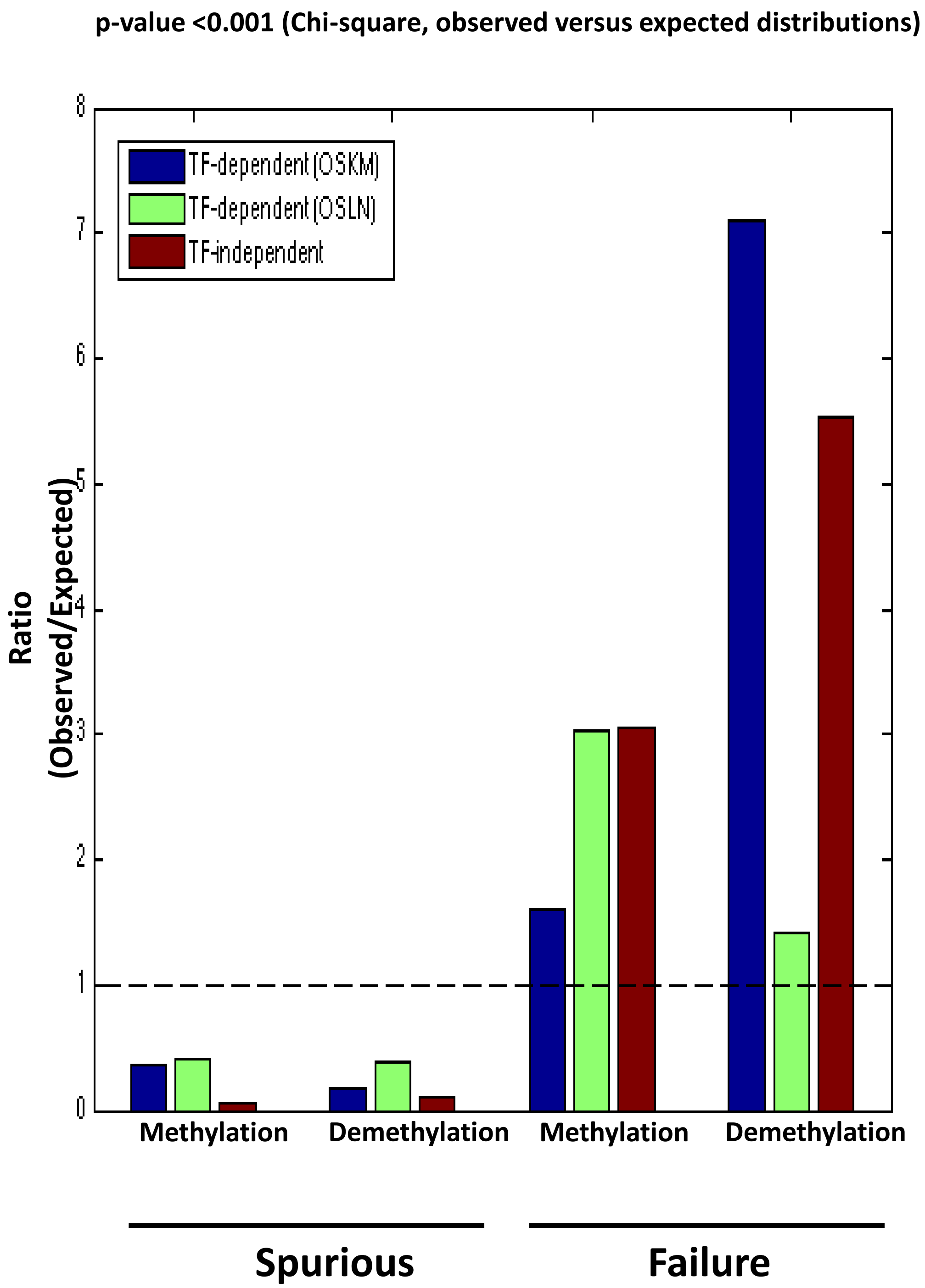

Figure S4 | **Observed versus expected ratio for the four classes of aberrations to the DNA methylation pattern**. Ratio between the observed number of probes in each class and the randomly expected number of probes based on the size of each group in Figure S3. Blue bars represent the four classes of aberration in Y-iPS cells; Green bars represent the T-iPS cells and the red bars represent aberrations that are common in both Y-iPS and T-iPS cells.

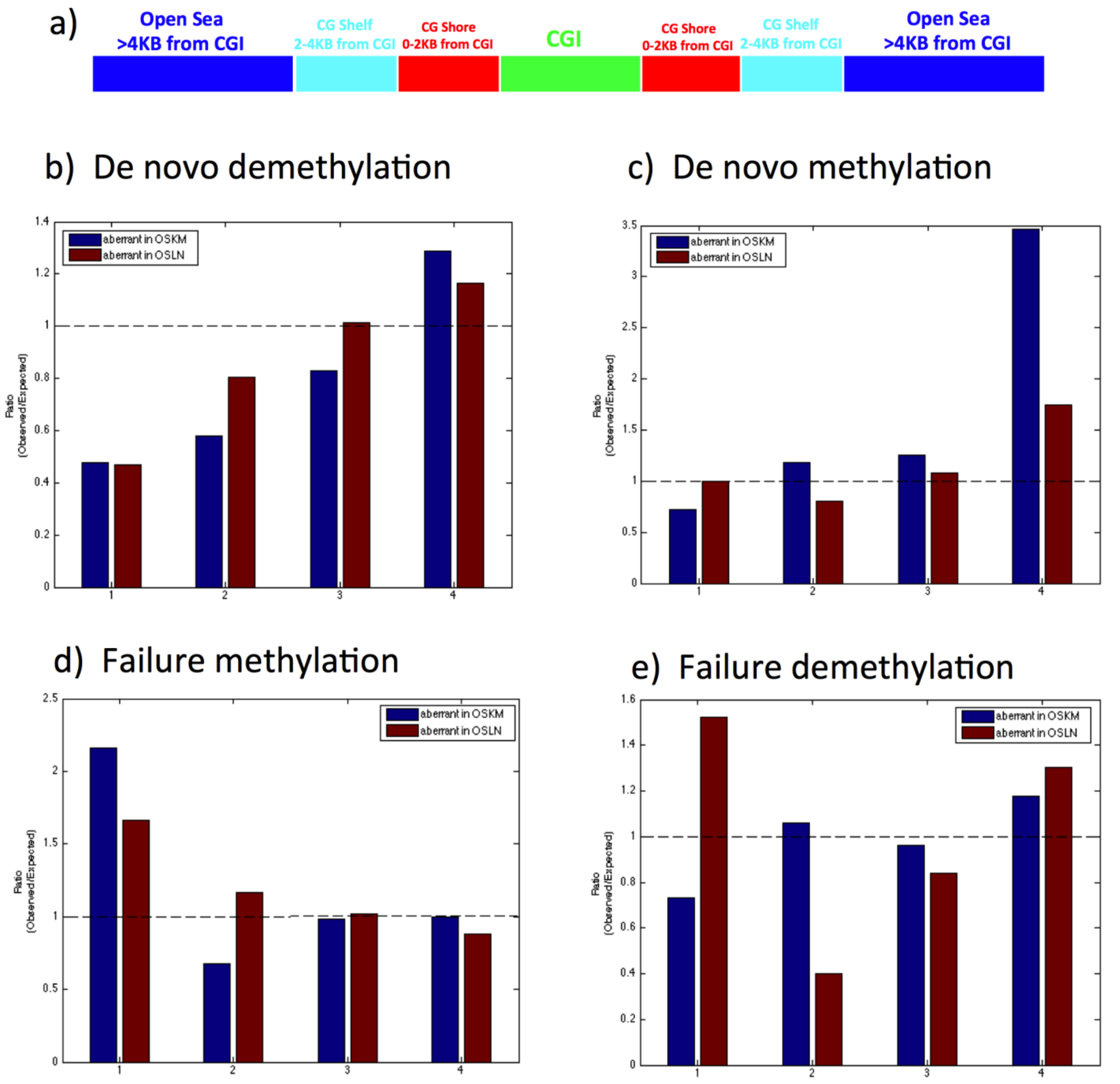

Figure S5 | **Observed versus expected ratio for the position relative to a CpG Island of each class of aberrations to the DNA methylation pattern.** (A) Schematic distribution of CpG islands, shores, shelfs and open sea. Ratio between the observed number of probes in the spurious demethylation class (B), spurious methylation class (C), failure to methylate class (D), failure to demethylate class (E) versus the randomly expected number of probes based on the size of each group in Figure S3. Blue bars represent aberration in Y-iPS cells; red bars represent aberration in T-iPS cells

a) Yamanaka-iPS specific aberrations

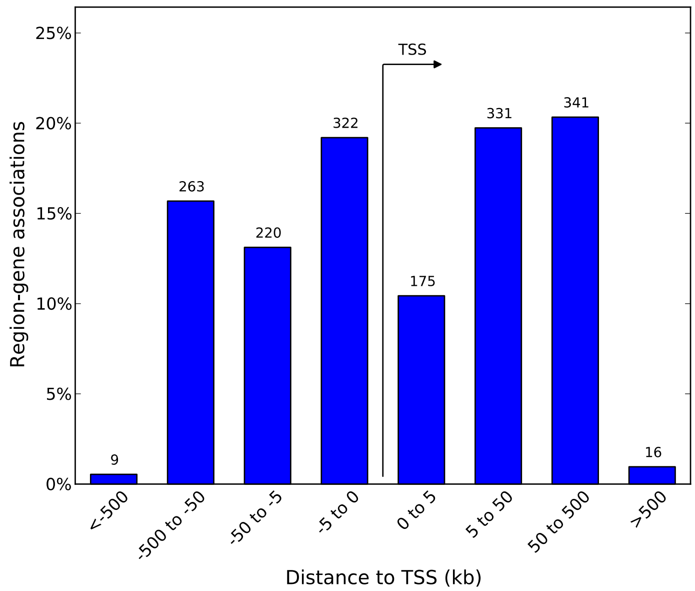

b) Thompson-iPS specific aberrations

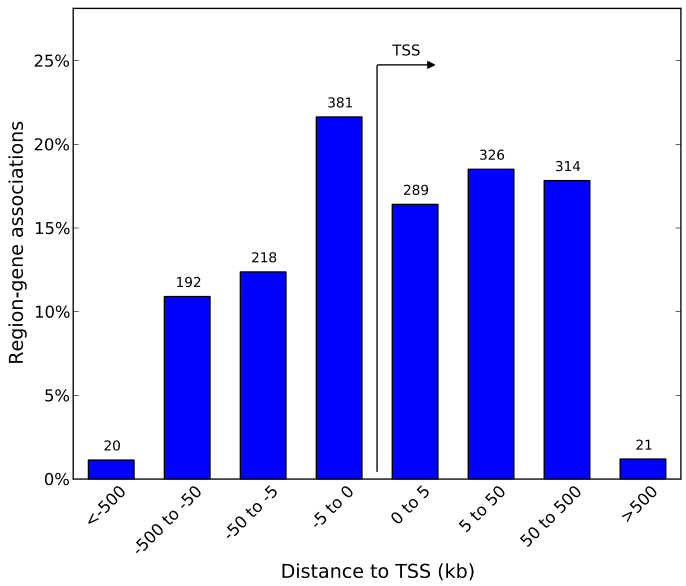

Figure S6 | **Region-Gene association graph for the position relative to a TSS of T-iPS and Y-iPS-specific aberrations to the DNA methylation pattern. Bined by orientation and distance to TSS.** (A) Yamanaka-iPS specific aberrations . (B) Thompson-iPS specific aberrations.

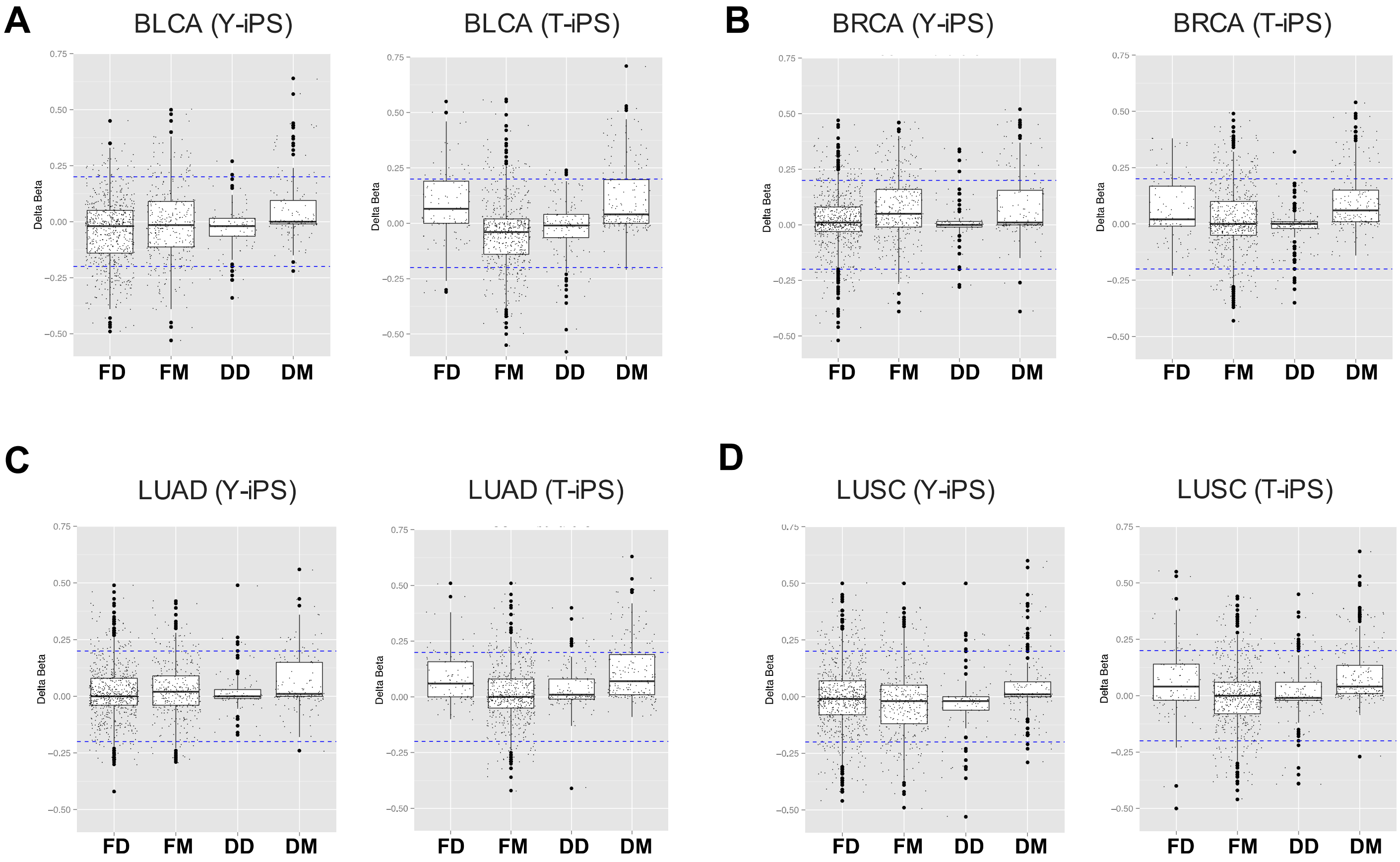

Figure S7 | **Cancer versus Normal profile of Y-iPS and T-iPS aberrantly methylated regions**. DNA methylation difference (Delta Beta value) between cancer patients and matched normal tissue of each CpG site identified as aberrantly methylated in Y-iPS cells (left panels) or T-iPS cells (right panels). The DNA methylation data was obtained from the TCGA depository (http://tcga-data.nci.nih.gov) for (**A**) Bladder Urothelial Carcinoma – BLCA (171 tumor samples and 19 normal samples), (**B**) Breast invasive carcinoma – BRCA (613 tumor samples and 97 normal samples), (**C**) Lung adenocarcinoma – LUAD (409 tumor samples and 32 normal samples) and (**D**) Lung squamous cell carcinoma – LUSC (252 tumor samples and 42 normal samples).
